# Results of neck-specific exercise for altered postural sway in individuals with chronic whiplash-associated disorders: a longitudinal case–control study

**DOI:** 10.1038/s41598-024-66176-w

**Published:** 2024-07-02

**Authors:** Anneli Peolsson, Hilla Sarig Bahat, Dmitry German, Gunnel Peterson

**Affiliations:** 1 Department of Health, Medicine and Caring Sciences, Unit of Physiotherapy, Linköping University, Linköping, Sweden; 2 Occupational and Environmental Medicine Centre, Department of Health, Medicine and Caring Sciences, Unit of Clinical Medicine, Linköping University, Linköping, Sweden; 3 Department of Physical Therapy, University of Haifa, Haifa, Israel; 4 Centre for Clinical Research Sörmland, Uppsala University, Eskilstuna, Sweden

**Keywords:** Whiplash injury, Neck, Spine, Chronic, Postural balance, Physiotherapy, Rehabilitation, Exercise therapy, Follow-up study, Outcome, Diseases, Health care, Medical research

## Abstract

Postural sway has not been investigated before or after a neck exercise intervention in individuals with chronic whiplash-associated disorders (WAD). The aim of the study was to investigate postural sway in individuals with chronic WAD grades 2 and 3: (a) compared with healthy matched controls at baseline; (b) after three months of neck-specific exercise and (c) to investigate the correlation between postural sway with self-reported dizziness during motion and balance problems/unsteadiness. This is a longitudinal prospective experimental case–control intervention study. Individuals with WAD (n = 30) and age- and gender-matched healthy volunteers (n = 30) participated. Postural sway was assessed using an iPhone application. Measurements were carried out at baseline, and for those with WAD a second measurement was performed at the three-month follow-up when neck-specific exercise intervention ended. The WAD group performed significantly worse than the healthy group in both pathway and ellipse area double stance eyes closed at baseline (main outcome), but not at the three-month follow-up. The WAD group significantly improved after rehabilitation in both pathway double stance eyes closed and pathway single stance eyes open. The correlation between postural sway and self-rated dizziness during motion and balance problems was low to moderate. One may conclude that postural sway was improved after a neck-specific exercise programme. The study results strengthen earlier findings that individuals with WAD have worse balance outcome when they have to rely on neck proprioception (eyes closed). The study results may be important for the development of improved rehabilitation methods for WAD.

## Introduction

Whiplash-associated disorders (WAD) is a disabling condition^1^ after an indirect neck trauma, a whiplash injury, often in connection with a car accident^2^. About half of those with WAD will experience chronic (≥ 6 months) pain and disability^3,4^, influencing their health, work and daily life^4–7^.

Research studies have shown increased fatty infiltration in WAD^8–10^, especially in the deep neck muscles which contain many proprioceptors that are important for maintaining the sensorimotor system and balance^11–13^. Furthermore, decreased neck muscle endurance, altered sensorimotor function, altered movement pattern and disturbed eye-neck control have been reported^14–24^. Pain and impaired neck muscle structure and function may be followed by disabling feelings of unsteadiness and/or dizziness with postural instability, and are often more pronounced in those with acute or chronic WAD^25–31^. However, it is insufficiently studied whether neck-specific exercise can improve postural stability in those with chronic WAD, and the results are contradictory. Postural re-education, or exercise of deep ventral and dorsal neck muscles including sensorimotor exercises and axioscapular muscles targeting the lower and middle trapezius, have been shown to be ineffective^32^. Neck-specific exercise can improve clinical balance tests and self-evaluated dizziness when combined with a behavioural approach^33^.

Postural sway is an aspect of static balance, and is the movement in the horizontal plane around the centre of mass in a standing position, based on the interplay among many factors such as orientation in space, movement strategies and sensorimotor processes^34,35^. Neck-specific exercise due to improved cervical intermuscular interplay and sensorimotor function^24,36^ may have an impact on postural sway in WAD, but to the best of our knowledge there is no physical intervention study in WAD investigating postural sway. This knowledge may be important for improved rehabilitation methods for WAD to reduce postural instability.

In chronic WAD grades 1 and 2, advice was just as effective as more comprehensive exercise programmes, but with only a small treatment effect^37,38^. However, patients with worse symptoms, as in WAD grade 3, were excluded. Individuals with WAD grades 2 and 3 have been shown to worsen compared with waiting list^39^; individuals with more severe WAD therefore need more attention from a physiotherapist. Neck-specific exercises have shown good results in pain and disability in WAD grades 2 and 3^5,6,15,17,33,40^, but—to our knowledge—postural sway has not been investigated.

The aim of the present study was to investigate postural sway in individuals with chronic WAD grades 2 and 3: (a) compared with healthy matched controls at baseline; (b) after three months of neck-specific exercise; and (c) to investigate the correlation between postural sway with self-reported dizziness during motion and balance problems/unsteadiness.

The hypothesis was that patients would improve in both pathway and ellipse area double stance eyes closed after the rehabilitation period, and that self-reported measures would be correlated to objective measurements.

## Methods

This is a longitudinal prospective experimental case–control intervention study. Data was collected from October 4, 2018 to December 16, 2021. Thirty patients were recruited from nine county councils in Sweden to investigate the efficacy of three months of neck-specific exercise (with internet support (NSEIT), four visits to a physiotherapist and an online programme; or neck-specific exercise (NSE) at a physiotherapy clinic, twice a week, optimally 24 times). NSEIT has previously been reported to be noninferior to NSE without significant differences between the groups regarding pain and self-reported neck disability^32–34^, confirming our initial intention to treat the intervention group as one in the present study.

The study was approved by the Regional Ethical Review Board in Linköping, Sweden (dnr 2016/135-31). Data is stored at the Medical Faculty at Linköping University, Sweden (for 10 years after publication of data, according to law), and is accessible for the researchers. The trial was registered before data collection started (Clinicaltrial.gov Protocol ID: NCT03664934, initial release 09/11/2018) and the protocol was published^40,41^. All experiments were performed in accordance with the Declaration of Helsinki and Swedish law. Patients were recruited through healthcare personnel, reports in newspapers, social media and the university’s website. Interested patients contacted the research team through the project website at Linköping University. After completion of a short digital survey, a research assistant (an experienced physiotherapist) conducted a telephone interview to ask about the patient’s medical history. If the patient fulfilled the study criteria at that point, an appointment was booked with a physiotherapist to take additional medical history and carry out a physical examination. If all the study criteria were met, patients signed a written informed consent form. Thereafter, patients completed a written questionnaire regarding background data, pain, physical and psychological function, and health, and had additional physical measurements taken, and a time for a postural sway assessment was agreed. Generally healthy individuals matched for age and gender were consecutively recruited through students and staff at Linköping University and Linköping University Hospital, and through advertising on social media.

### Participants

Study criteria, WAD group: The inclusion criteria for individuals with WAD were: chronic (> six months until five years since the accident) neck problems corresponding to WAD grades 2–3 (grade 2: neck symptoms and clinical findings, grade 3: as grade 2 plus additional neurological findings in a physical examination)^42^ verified by clinical examination, shown to have exhibited a 20 mm minimum average estimated level of pain in the last week based on the visual analogue scale (VAS)^43^ and a neck disability level higher than 20% on the Neck Disability Index (NDI)^44^, of working age (18–63 years), with access to a smartphone/computer and the internet, experienced neck-related symptoms within the first week following the injury, right-handed in addition to experiencing either equal-sided or dominant right-sided pain. Exclusion criteria for the WAD group were as follows: signs of head injury at the time of whiplash injury (amnesia before or after the injury, loss of consciousness, altered mental status such as confusion, disorientation, focal neurological changes such as changes in perceptions of smell and taste). Additional exclusion criteria were: previous fractures or dislocation of the cervical spine, a considerable degree of known or suspected physical pathology including myelopathy, spinal tumour, spinal infection, ongoing malignancy, cervical spine surgery and severe neck problems in their medical history which resulted in sick leave for more than a month in the year before the current whiplash injury, generalised pain currently occurring elsewhere in the body, other illness/injury that may prevent full participation in the intervention, lack of ability to either understand or write Swedish, increased risk of bleeding, obesity (body mass index; BMI > 35), contraindications of MRI such as claustrophobia, metallic foreign bodies, pacemaker, cochlea implant, nerve stimulator and pregnancy.

Study criteria for healthy controls were: age- and gender-matched healthy individuals without current or previous neck pain or disability (VAS < 10 mm, NDI < 5%) who feel healthy overall, without known diseases. Exclusion criteria for healthy controls were: earlier neck injury, recurrent neck pain, earlier treatment for neck pain, increased risk of bleeding, BMI > 35, contraindications of magnetic resonance imaging.

Thirty-two participants (24 female and eight male; mean age 40.6 years, SD 10.2) with chronic WAD grade 2 (16 individuals) and WAD grade 3 (16 individuals) and 32 controls, matched for age and gender, participated in the study (Table 1).

**Table 1:** Baseline characteristics of participants with whiplash-associated disorders (WAD) and healthy controls.

	N	WAD	N	Control	*P* value
Age, mean ± SD	31	40.6 ± 10.2	32	40.6 ± 10.4	0.99
Sex, female, n (%)	32	24 (75%)	32	24 (75%)	1.0
BMI, mean ± SD	32	24.0 ± 3.7	32	24.8 ± 3.5	0.34
Months since injury, mean ± SD	32	26.5 ± 13.3		NA	
WAD grade, grade III, n (%)	32	16 (50%)		NA	
Physical activity level, median (IQR)	31	3.0 (3.0 to 4.0)	32	4.0 (3.0 to 4.0)	0.04
Neck pain average last week (VAS 0–100), mean ± SD	32	44.4 ± 19.8	32	1.6 ± (2.8)	< 0.001
Neck muscle fatigue, mean ± SD	32	5.3 ± 2.25	32	0.2 ± 0.56	< 0.001
Neck disability (NDI percent scale), mean ± SD	31	37.7 ± 12.5	32	1.1 ± 1.6	< 0.001
Dizziness during motion (VAS 0–100), mean ± SD	32	32.5 ± 21.4		NA	
Balance problems (VAS 0–100), mean ± SD	32	24.3 ± 14.8		NA	

SD: standard deviation; VAS: visual analogue scale (100 mm, 0 = no symptoms and 100 = worst symptoms); WAD: whiplash-associated disorders grade III (neck pain, clinical musculoskeletal signs and neurological signs); physical activity level: 1 = inactivity, 2 = low activity, 3 = moderate activity, 4 = high activity; NDI: Neck Disability Index (0% = no disability, 100% = highest score for disability); dizziness during motion and balance problems: 100 mm, 0 = no symptoms and 100 = worst symptoms.

### Intervention for the WAD group

The intervention consisted of neck-specific exercise distributed in two different ways, NSEIT and NSE^40,41^, but handled as one neck-specific exercise group. Our definition of “neck-specific” is exercises specifically targeting the neck muscles. The groups were originally meant to include half of the patients from the NSE group and the other half from the NSEIT group, but due to the Covid-19 pandemic, recruitment to the NSE group was not available. Treatment was conducted by physiotherapists in outpatient care (primary healthcare and private practitioners). Patients were asked not to seek other healthcare, especially physiotherapy, for their WAD during the study period. To motivate the WAD group and make them feel safe, they were offered information about the whiplash injury mechanism, neurophysiological and neurobiological processes underlying chronic pain, the relevant neck muscle anatomy and function, why they should exercise the neck muscles, and handling pain relapses. Exercises were chosen from a written framework of exercises^40^. The exercise programme started with daily postural exercises in a sitting position and neck exercises in a supine position using eye movement, aiming to facilitate the ventral and dorsal deep neck muscles. Eye movement and posture have previously been shown to facilitate neck muscle activity^23^ and to improve cervical kinaesthesia after using the present programme^24^. The participants were informed that contracting the superficial neck muscles was not allowed, and were taught how to monitor this. Achieving a pattern of correct movement and being able to activate the deep neck muscles were crucial before gradual exercise progression. This progression started with slight manual resistance, still in a supine position, followed by exercises in a sitting position, both with and without manual resistance. As the last step, participants used rubber bands around their heads in a sitting position and exercises against gravity^40^ with the aim of increasing general neck muscle endurance^36^, involving both deep and superficial neck muscles^45^. The initial exercises aimed to improve the interplay of neck muscle layers^17^ between deep and superficial neck muscles, and to reduce pain and disability^40^. The exercise dosages were individually adjusted and progressively increased in dose and severity, and were guided to ensure correct performance. Pain provocation, other than muscle soreness, was not accepted. The participants could still perform the first exercises after progression. Details of the exercise programme—including photos, text, information provided and details of progression—can be found elsewhere in supplementary files 1 and 2^40^. The WAD group underwent a 12 week training programme. Upon the conclusion of the treatment period, the participants are encouraged to continue the exercises in their own time. Participants in both WAD groups received the same information and the same exercise programme. The NSEIT group received most of their information through the online programme (text, photos and videos of the exercise) via the university’s platform, and the NSE group received information from the physiotherapists at their twice weekly physiotherapy visits. Adverse events and harms were registered by the test leaders^40^.

### Postural sway

To be able to use postural sway as an evaluation method in clinical practice, the equipment had to be available, cheap to buy, easy to use and comfortable to wear. An iPhone application (for use on an iPhone to be attached to a belt at the pelvis (S2)^46^, and fulfilling the criteria above) was developed for measuring postural sway^46^. The study by German et al.^46^ showed that the directional bias, analysed by Bland–Altman plots, increased with the level of instability, i.e. the difference between devices in relation to the mean was greatest in single leg stance followed by tandem and double stance, strengthening the reliability of our findings, especially regarding double stance in the present study. Good to strong correlations were found between force plate and the customised smartphone application (CSA) for double stance of pathway and ellipse area (r = 0.79–0.85), and moderate to strong correlations for single leg stance (r = 0.53–0.84). Reliability was moderate to high for pathway in double stance and single leg stance (inter-class correlation [ICC]: 95%; confidence interval [CI] 0.77, 62–0.88) and moderate for the ellipse area (ICC: 0.52–0.53; CI 0.30–0.72)^46^. The iPhone sensors were pre-calibrated^46^.

**Figure 1:**
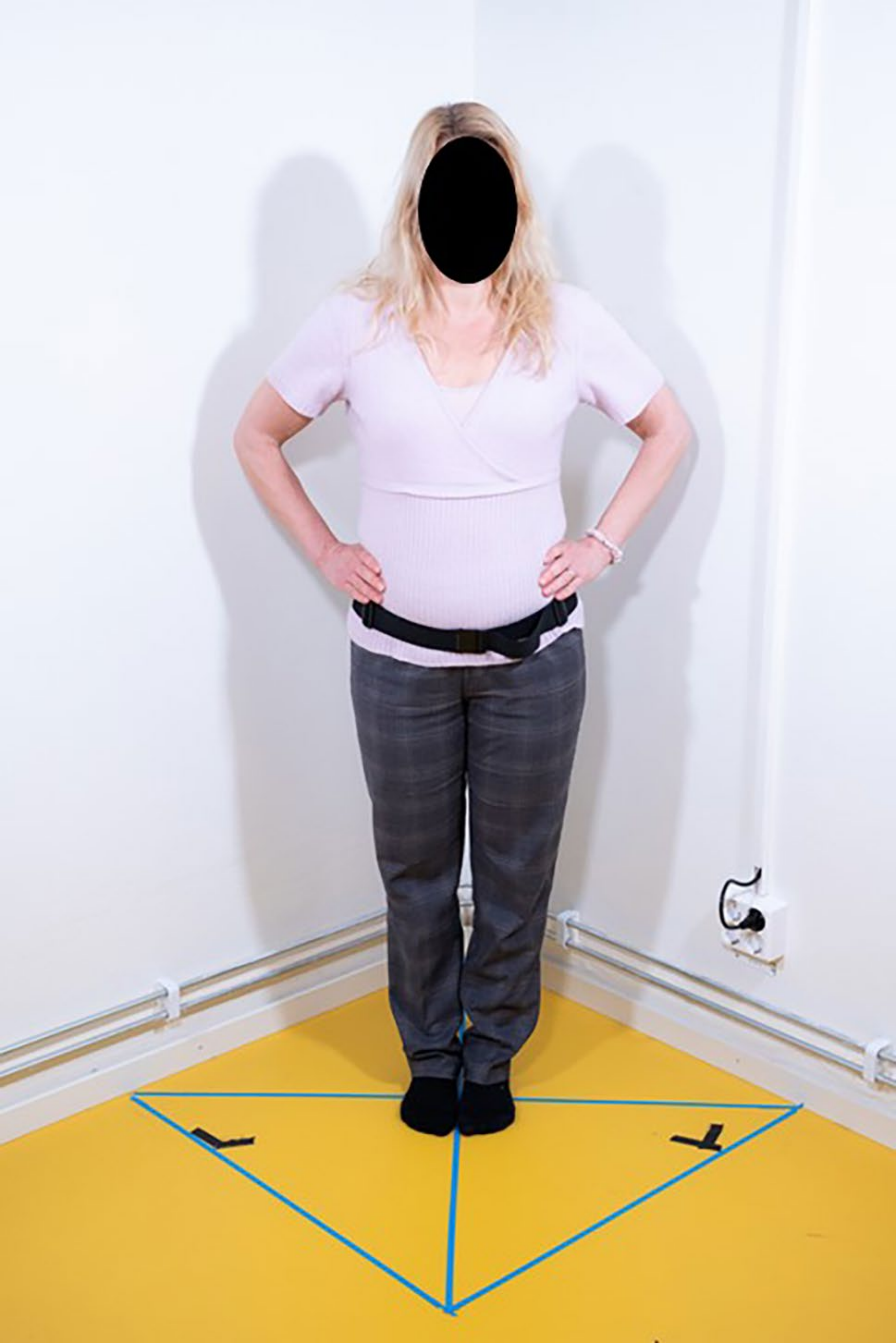
Double stance closed eyes.

**Figure 2:**
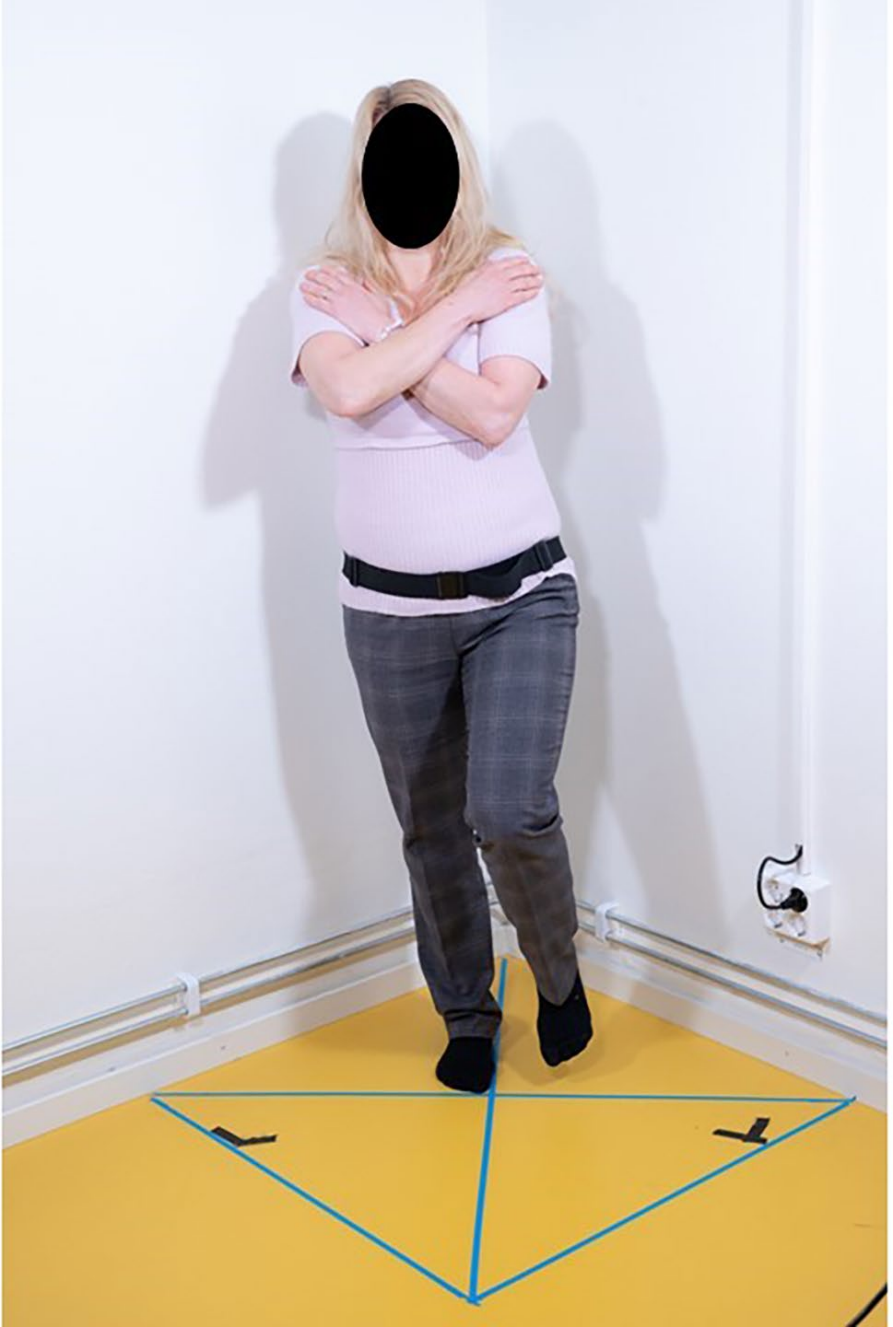
Single leg  open eyes.

In the present study, static balance was measured with postural sway. Postural sway analysis provided measures of pathway and 95% ellipsoid area in x and y planes^46^. Postural sway was assessed using an iPhone 8 customised application developed at the University of Haifa, Israel (in-house for research)^46^. The iPhone 8 was attached at the pelvis (iPhone sensors positioned at the centre of gravity, S2 level) with a waist belt^46^. Postural sway was assessed in two positions: double stance closed eyes (main outcome) (Fig. 1) and single leg open eyes (Fig. 2) (secondary outcome), both barefoot, each to be repeated three times.

Participants stood in a corner and the test leader remained within close reach to support and reassure the participant if needed. (There were no accidents/falls during the measurements.) The test leader carefully described and demonstrated the assessment procedure. Participants started with a 10 s warm-up session for both positions before measurements to ensure that they understand the instructions^46^. There was a 30 s rest in between repetitions, and a 60 s rest in between positions. Participants stood for as long as possible, up to a maximum of 40 (double stand test) or 45 (single leg test) seconds. Each session started with the app counting down from 5 to 1, followed by a beep to signal the start, and a beep to signal the end. The test leader did not encourage the participants during the tests.

Double leg stance, eyes closed: in double stance testing, both feet were on the floor, roughly hip width apart. Hands were on hips with eyes closed.

Single leg stance, eyes open: during the single leg stance the patient had free choice of preferred standing leg. The same leg was used at the follow-up measurement after intervention. The standing leg was kept straight. The raised leg was held in a slightly flexed hip and knee position. The raised leg was not allowed to touch the standing leg. Arms were crossed over the chest with eyes open.

Computerised data collection was initiated 5 s after the participant was in the testing position. Data analysis was performed on 20 retrieved seconds as in the study by German and Bahat^46^, showing that the customised smartphone application was valid and reliable.

Ethical issues should be addressed when using smartphones in medical research involving humans. In the present study, the iPhone was only used for the present purpose and was kept locked up at the university lab, and a coding number instead of the participant’s full name was used in the iPhone.

### Background data

The participants completed a baseline questionnaire prior to postural sway tests, asking about age, gender, body mass index, pain duration (months), neck disability (Neck Disability Index [NDI]: 0% = no disability, 100% = highest score for disability)^44,47^ and activity level (activity index: 1 = inactivity, 2 = low activity, 3 = moderate activity, 4 = high activity)^48^. The WAD group rated dizziness on average during motion and balance problems/unsteadiness experienced during the previous week measured with the Visual Analogue Scale (VAS) (100 mm, 0 = no symptoms and 100 = worst symptoms)^49^. WAD grade was recorded by the test leader.

Neck muscle fatigue was measured with the Borg CR-10 scale^50^ (0 = no fatigue, 10 = extremely strong fatigue) and neck pain intensity with VAS^51^ (0 = no pain, 100 = worst imaginable pain) before and after the postural sway test.

Compliance with exercise was self-reported at three-month follow-up (high compliance =  > 80%, medium compliance = 50–79% and low compliance =  < 50% compliance with the exercise). Eighteen of the participants (62%) reported high compliance with the exercise, seven reported medium compliance (24%) and four reported low compliance (14%).

### Statistical analyses

All data analyses were performed with SPSS statistical software, version 26. Demographic characteristics are presented as mean and SD or median and interquartile range (IQR), and analysed with Student’s *t*-test or Mann–Whitney U. For binary baseline data, χ2 tests were used.

Postural sway raw data was processed by a data analyst (Dmitry German; DG). Statistical analysis was performed (Gunnel Peterson; GP) in dialogue with a statistician.

Pathway of postural sway was calculated as the mean value of the three tests and as (a) the accumulated length of the tracked displacement during the test, (b) 95% ellipse area was calculated as the summed area within a 95% confidence ellipse of the centre of pressure and/or gravity of the samples recorded.

$$Pathway={\sum }_{i=1}^{n-1}\sqrt{({PSX}_{i+1}-{PSX}_{i}{)}^{2}+({PSX}_{i+1}-{PSX}_{i}{)}^{2}}$$

If one of the three test values was missing, the mean value of two tests was used, and if two were missing, one value was used in the analyses. In the WAD group, three individuals at baseline and two at follow-up had one missing value. One participant in the WAD group had two missing values at baseline and one missing value at the follow-up test. All missing values were due to inability to stand for as long as 20 s in the respective test.

Due to technical problems, data was missing from nine single leg stance and eight double stance tests for WAD participants at three-month follow-up.

The two sample groups were checked for normality using the Shapiro–Wilk test, showing non-normally distributed data (*p* < 0.05) except for double stance pathway at baseline in the control group (*p* > 0.05). Thus, all data was log transformed (Log^10^).

Differences at baseline between the WAD and control groups in postural sway (pathway and ellipse area) and neck pain were analysed with unpaired Student’s *t*-test. Differences between baseline and three-month follow-up in postural sway (pathway and ellipse area), neck pain and dizziness in the WAD group were analysed with paired Student’s *t*-test.

One participant in the WAD group and one in the control group were excluded from the analyses and were assumed to be outliers, likely due to registration failure (four times higher [WAD] or 21 times lower value [control] than the next value in the respective group).

Dizziness during motion and balance problems were correlated with pathway single leg and double stance and 95% ellipsoid area, and were evaluated with Pearson’s correlation coefficient. The strength of the correlation coefficients was based on Cohen’s scale^52^ (r < 0.3 = small, r 0.3 to 0.5 = moderate and r > 0.50 = large), interpreted with the revised range thresholds scale^53^: r < 0.20 = small, r 0.20–0.30 = medium, and r > 0.30 = large. The significance level for all tests was set at *P* < 0.05.

### Informed consent to publish photos

The woman in the photos is the first author, and she has given her informed consent to publish the photos.

## Results

### Comparison of postural sway in the WAD and control groups

There was a significant difference between the groups at baseline in postural sway double stance with eyes closed, showing greater pathway (7.0 cm, SD; 2.15, *p* = 0.003) and ellipse area (0.30 cm^2^, SD; 0.11, *p* = 0.006) in the WAD group (Tables 2 and 3). There were no significant differences between the groups in single leg stance, eyes open or single leg ellipse area (*p* > 0.13) (Tables 2 and 3).

**Table 2:** Postural sway, baseline and three-month follow-up values (mean ± SD) in the WAD and control groups, differences between WAD and control at baseline and three months, and within-group changes for the WAD group.

	N	WAD group baseline Mean ± SD	N	Control group (CG) baseline Mean ± SD	Between-group differences, WAD vs. CG baseline Mean (SE diff)	N	WAD group three months Mean ± SD	Between-group differences, WAD three months vs. CG baseline Mean (SE diff)	Change score WAD baseline to three months Mean ± SD
Pathway single leg, cm	31	39.27 ± 15.29	31	34,13 ± 17.69	5.1 (4.20)	22	33.06 ± 15.23	− 1.06 (4.66)	− 7.28 ± 18.70
Pathway double stance, cm	31	24.85 ± 10.78	31	17.84 ± 5.27	7.0 (2.15)	23	21.48 ± 12.82	3.6 (2.84)	− 4.25 ± 13.46
Ellipse area single leg, cm^2^	31	1.44 ± 2.24	31	1.52 ± 2.45	− 0.8 (0.60)	22	1.56 ± 2.68	0.04 (0.71)	− 0.10 ± 3.55
Ellipse area double stance, cm^2^	31	0.45 ± 0.58	31	0.15 ± 0.15	0.30 (0.11)	23	0.22 ± 0.17	0.07 (0.04)	− 0.24 ± 0.58

Mean ± SD: mean value and standard deviation.

Mean (SE diff): mean value (standard error of the differences between two means).

WAD: whiplash-associated disorders; CG: control group.

Between-group differences WAD three months vs. CG baseline: the WAD group’s three-month values were compared to the control group’s baseline values.

Between-group differences baseline and three months: positive values indicate higher value in the WAD group.

Within-group differences baseline to three months: negative values indicate decreased pathway and ellipse area at three-month follow-up in the WAD group.

**Table 3:** Log^10^ values in postural sway for the WAD and control groups at baseline and at three-month follow-up, and change score for the WAD group.

	N	WAD group Baseline Mean (95% CI)	N	Control group (CG) Baseline Mean (95% CI)	Between group differences, WAD vs. CG baseline Mean (95% CI)	*P* value	ES	N	WAD group three months Mean (95% CI)	Between group differences, WAD three months vs. CG baseline Mean (95% CI)	*P* value	ES	Change score WAD, baseline to three months Mean (95% CI)	*P* value	ES
Pathway single leg, cm	31	1.58 (1.51 to 1.65)	31	1.49 (1.41 to 1.56)	0.7 (-0.02 to 0.17)	0.131	.39	22	1.48 (1.40 to 1.56)	-0.01 (-0.11 to 0.10)	0.927	.03	-0.09 (-0.19 to -0.01)	0.037	.39
Pathway double stance, cm	31	1.37 (1.30 to 1.44)	31	1.23 (1.19 to 1.28)	0.12 (0.04 to 0.20)	0.003	.78	23	1.26 (1.20 to 1.32)	0.05 (-0.03 to 0.13)	0.238	.35	-0.09 (-0.17 to -0.01)	0.029	.32
Ellipse area single leg, cm2	31	0.33 (0.23 to 0.44)	31	0.29 (0.19 to 0.39)	0.01 (-0.12 to 0.14)	0.866	.04	22	0.30 (0.18 to 0.42)	0.01 (-0.14 to 0.15)	0.942	.02	-0.03 (-0.17 to 0.10)	0.593	.03
Ellipse area double stance, cm2	31	0.14 (0.08 to 0.20)	31	0.06 (0.04 to 0.08)	0.07 (0.02 to 0.13)	0.006	.73	23	0.06 (0.04 to 0.08)	0.02 (-0.01 to 0.05)	0.114	.45	-0.06 (-0.12 to 0.01)	0.057	.42

Mean (95% CI): mean value (95% confidence Interval).

WAD: whiplash-associated disorders; CG: control group.

Between-group differences WAD three months vs. CG baseline: the WAD group’s three-month values were compared to the control group’s baseline values.

Between-group differences baseline and three months: positive values indicate higher value in the WAD group.

Within-group differences baseline to three months: negative values indicate decreased pathway and ellipse area at three-month follow-up in the WAD group.

No significant between-group differences were found between the WAD groups’ three months test and the baseline tests for the healthy controls (*p* > 0.11) (Tables 2 and 3).

### Within subject effects in the WAD group from baseline to three-month follow-up

**Figure 3:**
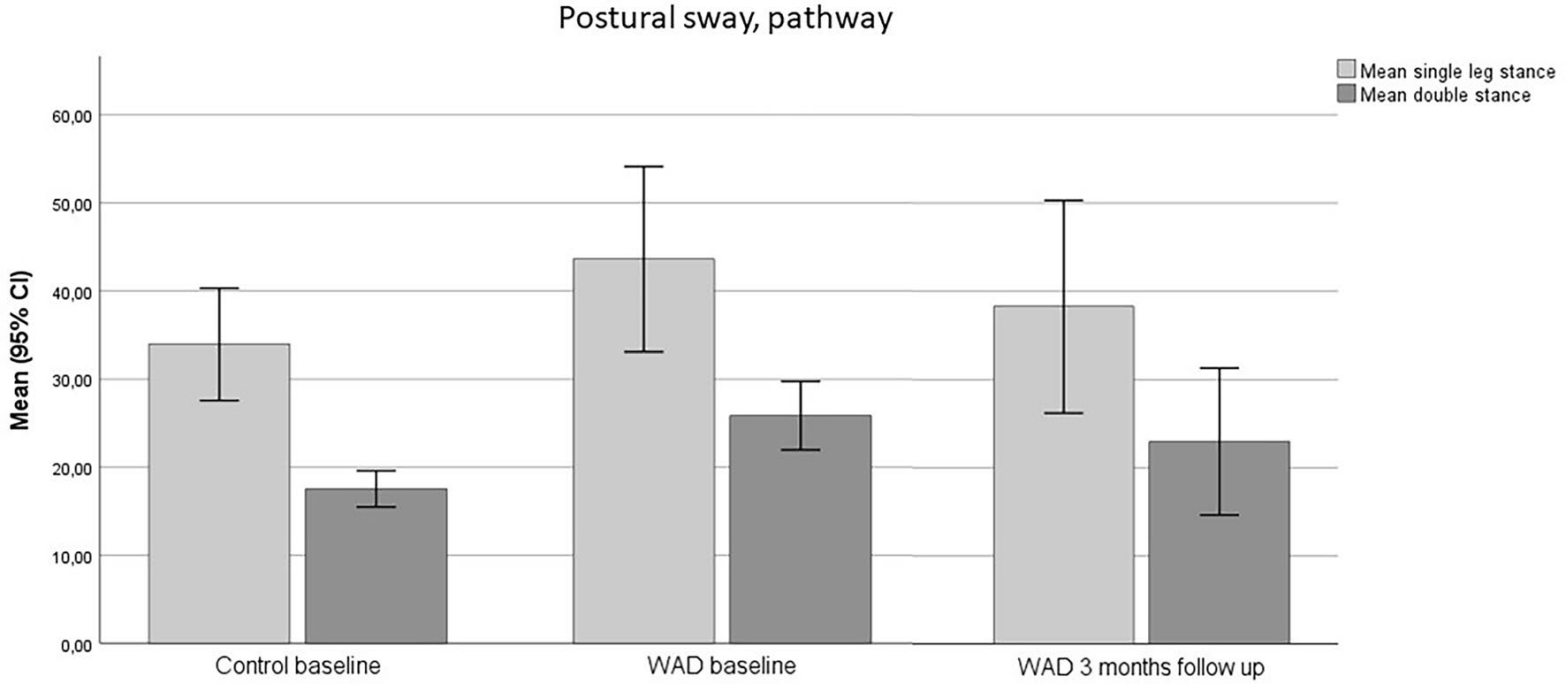
Pathway single leg open eyes and double stance closed eyes. Mean values and standard deviations (SD) at baseline in the control group: single leg (34.13, SD: 17.69) and double leg stance (17.84, SD: 5.27). Mean values and SD at baseline in the WAD group: single leg (39.27, SD: 15.29) and double leg stance (24.85, SD: 10.78). Mean values and SD at three-month follow-up in the WAD group: single leg (33.06, SD: 15.23) and double leg stance (21.48, SD: 12.82).

**Figure 4:**
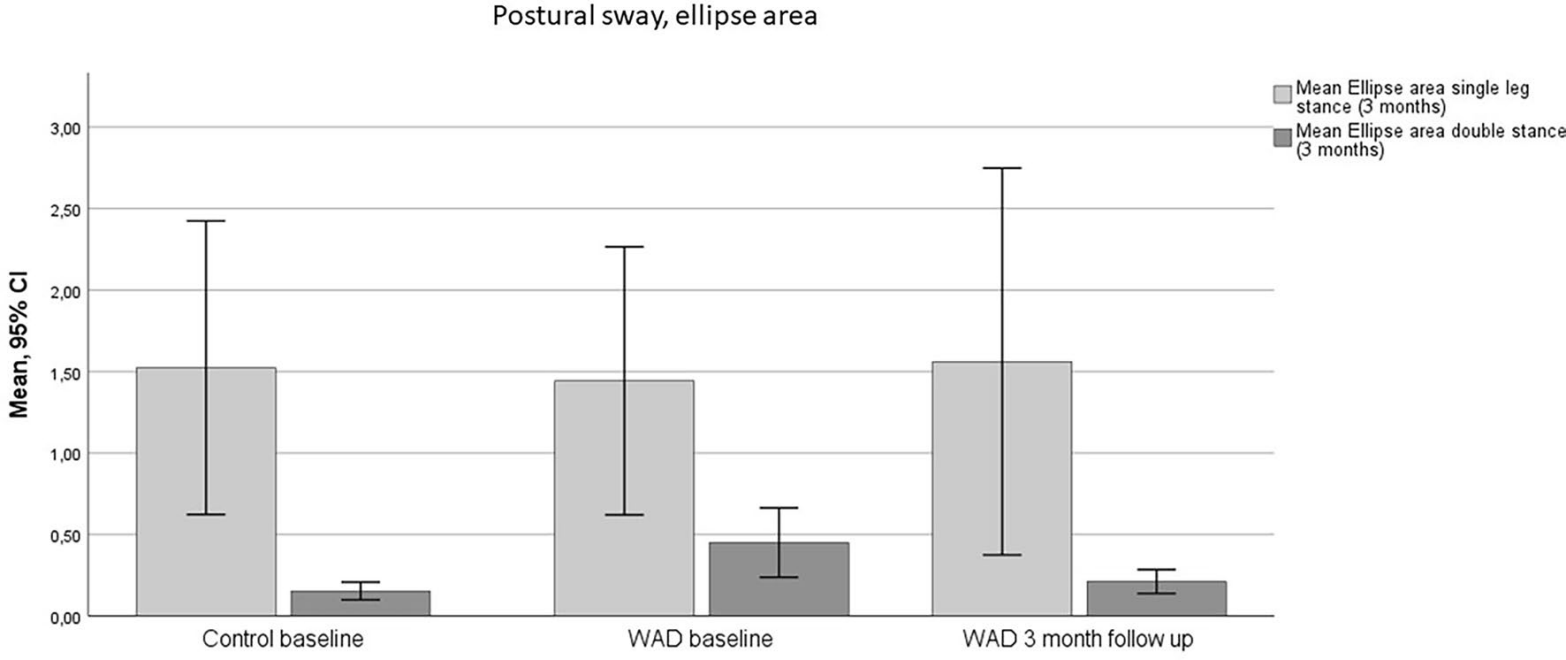
Ellipsoid area single leg open eyes and double stance closed eyes. Mean values and standard deviations (SD) at baseline in the control group: single leg (1.52, SD: 2.45) and double leg stance (0.15, SD: 0.15). Mean values and SD at baseline in the WAD group: single leg (1.44, SD: 2.24) and double leg stance (0.45, SD: 0.58). Mean values and SD at three-month follow-up in the WAD group: single leg (1.56, SD: 2.68) and double leg stance (0.22, SD: 0.17).

There was a significant improvement in the WAD group in pathway double stance closed eyes (− 4.25 cm, SD; 13.4, *p* = 0.029) and single stance (− 7.28 ± 18.7, *p* = 0.037) at three-month follow-up, where postural sway was decreased (Fig. 3, Tables 2 and 3). The ellipse area in double stance closed eyes also decreased (− 0.24 cm^2^, SD; 0.58), but the improvement was non-significant (*p* = 0.065) (Fig. 4, Tables 2 and 3).

### Correlation between self-reported dizziness, balance problems and the postural sway tests

Worse self-rated disturbed balance correlated with greater postural sway in the double leg stance (r = 0.39, *p* = 0.05) and single leg stance (r = 0.49, < 0.01), and with single leg ellipse area (r = 0.44, *p* < 0.01) in the WAD group. There was no correlation between dizziness during motion or any of the postural balance tests (r = − 0.08–0.27, *p* > 0.05).

## Discussion

The results show that individuals with WAD performed significantly worse than healthy controls in the main outcome double stance eyes closed at baseline. After a three-month rehabilitation period, the differences between the WAD and control groups were no longer significant in double stance eyes closed and the WAD group showed significant improvements over time. For single stance eyes open, there were no between-group differences at baseline or follow-up. However, the WAD group improved significantly after the intervention period.

Postural sway has been reported to be related to oculomotor dysfunction in those with WAD^54^. Individuals with WAD grades 2 and 3 have altered neck muscle function compared to heathy controls^15,24,36^. They have lower neck muscle endurance^36^, altered intermuscular interplay in the neck with less activation of the deep neck muscles and less synchronization between the deep and superficial muscle layers^15^, and impaired sensorimotor function^24^. Moreover, fatty infiltration in the neck muscles (contractile muscle tissue replaced with fat) has been reported in WAD^8–10^, especially in the deep neck muscles which contain many proprioceptors that are important for maintaining the sensorimotor system and balance^11–13^. Individuals with WAD who had a disturbed neck proprioception had to rely on visual feedback for balance performance. This has previously been shown for both WAD and other neck conditions^21,25,55^, and was confirmed in the present study where the WAD group had obvious difficulties in postural sway when they had their eyes closed, but not when they had their eyes open compared to healthy controls. There is sparse information regarding the effectiveness of neck-specific exercise programmes aiming for increased sensorimotor control, and neck muscle endurance to improve balance performance, including postural sway for those with WAD. Neck pain and disability, neck muscle endurance, neck intermuscular interplay and cervical kinaesthesia are all factors reported to be improved after a similar or the same neck exercise programme as in the present study^5,17,24,36^, which may be factors of importance for the improved postural sway in the present study. Treleaven et al.^33^ reported some effects of deep and superficial neck-specific exercise on clinical balance tests (measured in seconds or number of wrong steps) in a randomised controlled study, but mainly in the group that received a combination of neck-specific exercise and a behavioural approach. In the present study, virtually the same neck-specific exercise programme was used as in the study by Treleaven et al.^33^, but with two additional ventral neck muscle endurance exercises included, which seems to have been successful not only to improve ventral neck muscle endurance (unpublished data), but also to reduce postural sway double stance. Although the effectiveness on postural sway after a neck-specific exercise programme had to be investigated in RCTs, the results of the present study are promising as chronic WAD grades 2 and 3 have been reported to be disabling conditions influencing the whole of life^1,3,4,7^, and to be rather resistant to treatment^1,56–58^. Despite no significant differences between the healthy group and the WAD group in single leg stance eyes open, the WAD group improved after intervention, confirming existing knowledge in healthy individuals that sensorimotor disturbance and muscle fatigue are of importance for balance^59^ and may be improved after an exercise programme including these elements. Even in chronic neck pain patients, postural sway increased after experimentally induced neck muscle fatigue^60^.

To our knowledge, postural sway after an exercise programme has not been studied before in WAD grade 2 or 3. Eriksson et al.^61^ showed that both individuals with WAD grades 2 and 3 and patients with non-traumatic neck pain, but not healthy controls, had improved postural sway in an experimental situation where an intraoral appliance was used to improve temporomandibular sensorimotor function. Once again, the results show the importance of good sensorimotor function.

There was a not unexpected significant correlation between postural sway and self-perceived dizziness during motion and balance problems, although the correlation was moderate. The measurements were carried out in an experimental situation in two different tasks while patients probably rate situations during their daily life, for example when going to the grocery store. Psychological factors such as anxiety and coping, as well as present pain, may also influence the self-ratings. We can only speculate about why the self-reported correlation was slightly higher for single leg stance, eyes open than for double stance, closed eyes. One answer may be that it is more obvious for the individual in daily life, e.g. when putting socks or trousers on when standing on one leg if they have balance difficulties, or when walking in the dark.

One aspect of balance is postural sway, which is recommended in a meta-analysis by de Zoete et al.^62^ as clinically useful in individuals with neck pain. The present study provides further understanding of postural sway in chronic WAD. Future studies investigating the combination of neck-specific exercise and balance exercise or oculomotor muscle exercise would be useful.

The participants in the present sub-group^41^ of the RCT by Peterson et al.^40^ are a representative sample of the whole RCT group regarding age, gender, BMI, pain duration, neck pain and NDI. However, in the present study half of the participants had WAD grade 3, compared to one third in the RCT—in other words, a larger number of participants here with a more severe WAD grade with neurological symptoms included. We can only speculate about the reason for this, but one theory is that those with more severe WAD grades are more eager to participate in additional investigations. A higher number of those with WAD grade 3, often regarded as difficult to treat and often excluded from rehabilitation studies, strengthens the present results even more in suggesting that neck-specific exercise may improve postural sway. In earlier studies, individuals with WAD grade 3 have often been excluded.

### Limitations and advantages

Previous data from which to count the sample size was not available, and there was no earlier knowledge of the effect on postural sway after a neck-specific exercise period for those with longstanding pain after a whiplash injury. The study may be seen as a pilot study. However, our earlier studies have shown that 30 individuals are enough for comparison between individuals with health problems and healthy controls. Nevertheless, it may be that the sample size was too small for the single stance. Further larger RCTs including a patient control group will be needed to draw firm conclusions.

Postural sway is one important aspect of balance needed for daily life, but may need to be supplemented with other more dynamic measures such as walking in a figure of eight and walking when turning the head. Measurements may also be needed on even surfaces.

Some technical competence is needed to be able to process the data, although this was dealt with by a physiotherapist in the present study. An internet connection is needed to transfer data, but may limit the location where data is transferred.

This study did not investigate the effectiveness of different rehabilitation protocols, but the study aims and scope was limited to evaluating whether a specific protocol could be evidenced to be effective.

Advantages include no prior calculation being needed when using the application for postural sway measurements, the WAD group having confirmed WAD grades 2 and 3 through detailed medical history and a clinical examination, and the healthy group being matched regarding age and gender. Another advantage is that the WAD group was treated in outpatient care by several physiotherapists (after careful instructions) over a large geographical area, increasing generalisation and opportunities for implementation on a larger scale.

## Conclusions

The WAD group performed significantly worse than the healthy group in the main outcome double stance eyes closed at baseline, but not at follow-up as the WAD group improved after rehabilitation as hypothesized. Single leg stance eyes open also improved after the intervention. This strengthens earlier findings that individuals with WAD have worse balance outcomes when they have to rely on neck proprioception (eyes closed). The results showed that postural sway could be improved after a neck-specific exercise programme. The study results may contribute to the development of improved rehabilitation methods for WAD.

## Data Availability

Data will be made available upon reasonable request and after ethical permission.
